# Gut microbiota and BMI throughout childhood: the role of firmicutes, bacteroidetes, and short-chain fatty acid producers

**DOI:** 10.1038/s41598-022-07176-6

**Published:** 2022-02-24

**Authors:** Timothy A. Houtman, Henrik A. Eckermann, Hauke Smidt, Carolina de Weerth

**Affiliations:** 1 Behavioural Science Institute, Radboud University, Nijmegen, The Netherlands; 2 Donders Institute for Brain, Cognition and Behavior, Department of Cognitive Neuroscience, Radboud University Medical Center, Nijmegen, The Netherlands; 3 Laboratory of Microbiology, Wageningen University and Research, Wageningen, The Netherlands

**Keywords:** Predictive markers, Microbial communities, Microbiome, Risk factors

## Abstract

Childhood obesity is a risk factor for numerous health conditions. A critical factor in the etiology of obesity appears to be the gut microbiota, which is the microbial community that resides in the human gut. The ratio of the phyla Firmicutes and Bacteroidetes (F/B) and gut bacterial genera that produce short-chain fatty acids (SCFA) have been suggested to contribute to obesity. The current study investigated (1) whether differences in F/B ratio can be observed in infancy and childhood in relation to zBMI in healthy children, and (2) whether an innovative proxy measure adds evidence to a relationship between SCFA producers and the etiology of obesity. Stool samples were collected at five time points, and zBMI was assessed at eight time points throughout the first 12 years of life. Our confirmatory analyses with Bayesian multilevel models showed no relationship between the F/B ratio and zBMI. Also, a proxy measure constructed from known SCFA producers was unrelated to zBMI throughout the first 12 years of life. Exploratory analyses using multilevel and random forest models suggest that the relative abundances of Firmicutes and Bacteroidetes were independently negatively associated with zBMI from infancy through childhood, and the SCFA producing genera Subdoligranulum and Alistipes were negatively related to future BMI in childhood.

## Introduction

In the last decades, the worldwide prevalence of childhood obesity has reached epidemic proportions^1^. Childhood obesity is accompanied by numerous short- and long-term health consequences such as an increased risk of metabolic and cardiovascular conditions as well as a higher likelihood of psychological comorbidities during childhood and adulthood^2–4^. It has been firmly established that diet and lifestyle choices are associated with changes in weight and body composition^1,5^. However, biological, behavioral, and environmental factors, and their interactions, may also drive individual variation in proneness to obesity^1^. One such factor is the gut microbiota. The gut microbiota, which is the complex community of predominantly bacteria, but also including archaea, microeukaryotes and viruses, that resides in the gut, is believed to play an essential role in many aspects of physiology, such as energy homeostasis, blood circulation, and immunity^6–8^. Previous research has shown that disruptions to the gut microbiota by external factors such as diet and lifestyle, but also delivery mode and antibiotic use, may predispose individuals towards weight gain^9–11^.

Early reports comparing obese animals and humans to lean counterparts described a differential gut microbiota composition between them^12,13^. Obese mice and humans exhibited an increase in the relative abundance of the phylum Firmicutes and a decrease in the phylum Bacteroidetes, which was reversed after diet-induced weight loss^13,14^. It was suggested that the difference between lean and obese individuals can be attributed to functional characteristics of the gut microbiota. The increased Firmicutes to Bacteroidetes (F/B) ratio and change of body composition could reflect an increased capacity to ferment dietary polysaccharides to short-chain fatty acids (SCFA). SCFAs such as butyrate, propionate, and acetate, partake in various physiological processes and are believed to play an important role in gut-barrier functioning and appetite regulation^15^. Moreover, the phylum Firmicutes includes many known SCFA producers^16,17^ and SCFAs provide an estimated 10% of the total dietary energy supply in humans^18^. Based on these early findings, it was theorized that the gut composition of obese individuals may include higher abundancies of species specialized in energy harvesting.

Subsequent studies have replicated the increased F/B ratio in relation to obesity in obese animals and adult humans^19–21^, whereas others have reported an inverse or no relationship between obesity and F/B ratio^22–24^. Fewer studies have investigated the F/B ratio in children. One systematic review about the F/B ratio in relation to obesity in children between the ages of 3 weeks and 13 years found comparable results to those in adults^25^. However, these studies' evidence is limited because most of them did not assess the F/B ratio at phylum level but rather changes in lower order taxa that belong to either phylum^26,27^. To our knowledge, only three other studies, that were not included in the previously mentioned review, cross-sectionally assessed the phyla Firmicutes and Bacteroidetes as a whole in children in relation to BMI. Goffredo et al.^28^ found that F/B ratio was significantly and positively associated with BMI and body fat composition in 84 US children and adolescents between ages 9 and 18. Another study by Xu et al.^29^ included 175 Kazakh children between age 7 and 13. They found a negative relationship between the Bacteroidetes to Firmicutes ratio (B/F ratio, inverse of F/B ratio) and BMI, which is in line with the initial results in mice and adults. In contrast, Mbakwa et al.^30^ found no relationship between B/F ratio and BMI in a larger sample of 295 Dutch children between the ages of 6 and 7. Overall, these data suggest that there is no clear evidence to support the notion that obese and lean gut compositions differ from each other or whether the F/B ratio could be a biomarker for the development of obesity in children.

Other research investigating the link between SCFAs and body composition has found that obese adults have a higher SCFA concentration in stool samples of compared to lean counterparts^23,31,32^. One study found that butyrate, propionate, and acetate concentrations in plasma had a stronger positive association with body fat composition compared to F/B ratio in children between 9 and 18 years^28^. Interestingly, F/B ratio was found not to be correlated with SCFA in stool or plasma samples^28^. These findings suggest that a gut microbiota directed towards SCFA production rather than simply an increased F/B ratio may be able to absorb more energy from one's diet and, in some instances, lead to the development of a higher BMI.

Despite evidence suggesting a link between F/B ratio, SCFA producers, and weight gain, contrasting results have also been reported and anti-obesity effects have also been observed in relation to SCFAs^33,34^. Various studies in mice have shown that acetate is associated with reduced appetite, adipose tissue reductions, and improved glucose tolerance^35,36^. Furthermore, dietary SCFA supplementation led to reductions in body weight and improved insulin resistance in mice without altering dietary patterns or physical activity levels^37^. In humans, targeted delivery of propionate to the colon of obese individuals resulted in a significant reduction of appetite, body weight, and adiposity, as well as improved insulin sensitivity^38,39^. These findings suggest a more complex relationship between the gut microbiota and obesity than solely an increase in dietary energy supply.

Based on the evidence thus far, it is unclear whether F/B ratio and SCFAs can be considered biomarkers for obesity or not. The F/B ratio and SCFAs are often associated because most butyrate producers are affiliated with the phylum Firmicutes^40^. However, the Firmicutes contain many more species that do not have the capacity to produce SCFAs. As such, the F/B ratio may not represent the functional difference proposed in the energy harvesting hypothesis. Furthermore, most previous studies have focused on animal and human adult studies or cross-sectional relationships. The current study investigates the validity of the F/B ratio and SCFA producers in relation to BMI in a developmental context. In addition to studying the energy harvesting hypothesis in a developmental framework, we also developed a new proxy measure that uses an aggregated score of genera known to be capable of producing SCFAs as a variable of interest. Bayesian multilevel modeling and Random Forests were used to study the (longitudinal) relationship between the gut microbiota and BMI from birth to 12 years of age in a healthy Dutch community sample. BMI was assessed at eight time points, and stool samples were collected at five of these time points. Despite conflicting evidence, we predicted, on the basis of the energy harvesting hypothesis, to find positive associations between BMI and both F/B ratio and SCFA producers, both cross-sectionally and with future time points.

## Methods

### Participants and procedure

The study is part of a longitudinal study called BIBO (Basale Invloeden op de Baby Ontwikkeling) that has been ongoing for over 14 years^41^ and which was granted ethical approval by the Faculty Ethics Committee of Radboud University (ECG 300107, ECG 22111/130112) in accordance with the Declaration of Helsinki. Initially, 193 healthy mother-infant pairs were recruited towards the end of the pregnancy in the Nijmegen, Arnhem, and surrounding areas in the Netherlands. Mothers of the participants gave written informed consent before starting the study. Over the years, participants dropped out for different reasons, such as lack of time or personal reasons. At the data collection wave at age 12 years, 160 children (47% girls) were still active in the study. The weight and length of children were obtained at 1, 3, and 4 months of age and 2, 6, 7, 10, and 12 years of age. Fecal samples were obtained from children at 1, 3, and 4 months of age and 6 and 10 years of age. The means and standard deviations of the exact age of collection in days can be found in Table 1. The fecal samples were collected by participants at home and stored in their home freezer before being transferred to the lab facilities and stored at a temperature of −80 degrees °C.

**Table 1:** Age of data collection in days.

Variable	1 month		3 months		4 months		2 years		6 years		7 years		10 years		12 years	
	M (SD)	*n*	M (SD)	*n*	M (SD)	*n*	M (SD)	*n*	M (SD)	*n*	M (SD)	*n*	M (SD)	*n*	M (SD)	*n*
BMI	33.1 (7.5)	143	92.5 (9.7)	148	128.5 (11.2)	146	2.17 (0.25)	168	6.1 (0.24)	144	7.0 (0.18)	157	10.1 (0.30)	156	12.7 (0.30)	98
Stool sample	28.3 (3.3)	135	84.9 (14.8)	121	115.3 (14.6)	119			6.1 (0.15)	129			10.1 (0.25)	142		

The exact day of collection for some stool samples (12.9%) was not recorded but still utilized in the analyses. All means and standard deviations of 2 years and onwards are reported in years rather than days.

### Measurements

#### BMI

BMI z-scores (zBMI) were calculated with the package zscorer^42^ using length (in cm), weight (in kg), age (in days), and sex. Zscorer adjusts BMI (kg/m^2^) for age and sex according to the WHO Child Growth Standards resulting in values between 3 and −3 that correspond to the 99.865th and 0.135th percentiles respectively. This guideline was developed to provide an international standard for children's growth and development^43^. There were potential measurement or entry errors for the length or weight variable for six children, which resulted in zBMI far above 3 or below −3. These values were removed from the analyses. The inclusion of outliers did not change any conclusion of reported analyses unless reported otherwise.

#### 16S rRNA gene sequence data

DNA was extracted from fecal samples as described before^44^. 16S ribosomal RNA (rRNA) gene amplicons spanning the V4 variable region were amplified using primers and conditions as reported earlier^44^. PCR products were purified, pooled in equimolar amounts with positive and negative control samples, and sent for sequencing on the Illumina platform at Eurofins Genomics, Germany. Raw sequence data was then processed and further analyzed using the NG-Tax pipeline for barcode-primer filtering, demultiplexing, and taxonomic assignment^45,46^. NG-Tax is a highly accurate and well-validated pipeline for 16S rRNA gene amplicon sequence analysis. For each sample, an amplicon sequence variant (ASV) table was created with the most abundant sequences identified, and a user-defined minimum relative abundance threshold was used. In this study, the minimum relative abundance threshold was set to 0.1%. Taxonomy was assigned to ASVs using the Galaxy implementation of NG-Tax^46^ and the Silva 132 SSU Ref database.

#### Birth weight

Because birthweight has been associated with the development of obesity in later life (see systematic review by Baidal et al.^47^, birthweight was standardized and added as a covariate in the model.

### Statistical analysis

All analyses were performed in the statistical programming language R^48^ version 4.0.2.

#### F/B ratio

The 16S RNA gene sequence data was first imported using the package phyloseq^49^. Like previous studies^50,51^, the F/B ratio was calculated by dividing the relative abundances of Firmicutes by the relative abundance of the Bacteroidetes.

#### SCFA producers

The SCFA producers were chosen based on literature and those bacteria that have the genetic potential to produce SCFAs^52–54^. The known SCFA producers were chosen at the genus level (Table 2). We are aware of the fact that this might be an oversimplification of the actual SCFA production potential, considering that not all members of a given genus necessarily have the same metabolic capacity, and acknowledging that not all SCFA producing taxa have been characterized to date. Nevertheless, current knowledge of the microbial taxa involved in SCFA production is sufficiently sound for the generation of proxies such as described here. To calculate the final sum score of SCFA producers, we first added up the raw counts of all SCFA producers and replaced the separate SCFA producers with a single sum score for each sample. We then applied the centered-log-ratio (CLR) transformation to all samples in order to make the data compatible with conventional statistical methods^55^ using the package phyloseq.

**Table 2:** Names of SCFA producers and their metabolites.

Phylum	Genus	SCFA produced
Actinobacteria	Bifidobacterium	Acetate
Bacteroidetes	Alistipes	Propionate
Bacteroidetes	Bacteroides	Propionate
Bacteroidetes	Prevotella	Propionate
Firmicutes	Anaerostipes	Butyrate
Firmicutes	Blautia	Propionate
Firmicutes	Coprococcus	Propionate and butyrate
Firmicutes	Dialister	Propionate
Firmicutes	Eubacterium hallii group	Propionate and butyrate
Firmicutes	Eubacterium rectale group	Butyrate
Firmicutes	Faecalibacterium	Butyrate
Firmicutes	Holdemanella	Butyrate
Firmicutes	Phascolarctobacterium	Propionate
Firmicutes	Roseburia	Propionate and butyrate
Firmicutes	Subdoligranulum	Butyrate
Proteobacteria	Desulfovibrio	Acetate
Verrucomicrobia	Akkermansia	Propionate and acetate

#### Confirmatory analyses

The cross-sectional relationships were investigated by constructing a Spearman correlation matrix including the F/B ratio and zBMI data at all time points. The same matrix was constructed with SCFA producers and zBMI data at all time points. The Benjamini–Hochberg procedure^56^ was applied to both matrices to control for multiple testing.

To investigate longitudinal relationships, we fitted multiple Bayesian linear multilevel models to estimate the association between zBMI (T) and F/B ratio of the previous time point (T − 1) or the sum score of the SCFA producers (T − 1), respectively, controlling for birthweight and zBMI (T − 1). The covariate structure was the same across all models, and for all models, we allowed the intercept and slope of our effect of interest (SCFA sum/FB ratio) to vary. The models were fitted using the R package brms^57^, which utilizes the Hamiltonian Monte Carlo (HMC) Markov chain Monte Carlo (MCMC) algorithm implemented in Stan^58^ to estimate parameters. Before model fitting, we applied multiple imputations using predictive mean matching (m = 100, iter = 40) as implemented in the mice package^59^. We used the imputation algorithm with additional variables, including length, weight, and age, to allow the highest possible precision of imputations. The chosen number of imputations is necessary to ensure proper estimation of the tails of the posterior distributions of the β coefficients of the predictors according to the recommendations summarized in van Buuren^60^. Conclusions between imputed and non-imputed data did not differ unless otherwise reported.

All variables were standardized before fitting the model to ease interpretability, model convergence, and prior specification. Values of zBMI did not require standardization as they were already standardized. Prior specification was based on prior predictive simulations while we used a normal prior (M = 0, SD = 0.5) on our parameters of interest across all models. For specific exploratory models, where we included the separate SCFA producers as predictors, we used a prior with a slightly narrower shape (M = 0, SD = 0.25) instead. The priors for the β coefficients are only slightly regularizing and could very quickly become overwhelmed if there is a signal in the data. Across all models, the prior for the standard deviation of the varying intercept and slope was set to an exponential prior with a rate of 1. For two exploratory models, we had to use a stronger prior with a rate of 15 to reach proper convergence without divergent transitions. The models were fitted with 10.000 iterations, and the control setting adapt_delta was set to 0.99 to prevent few divergent transitions from occurring in some of the models. After model fitting, we first ensured proper chain convergence by inspecting the chain plots. This was followed by posterior predictive checks to assess appropriateness of the model for the data. It was ensured that all models converged properly before interpretation.

#### Exploratory analyses

The random forest (RF) algorithm allows a data-driven approach that can identify more complex interactions of predictor variables and nonlinear effects compared to linear models. Furthermore, the provided feature importances can be utilized for biomarker discovery^61,62^. We used the R package ranger^63^ to predict zBMI scores from genus level relative abundances. We fitted two RF models for each time point: One model predicting the future zBMI and one cross-sectional model. Each model was performed as follows: First, hyperparameter tuning of the parameters mtry and sample.fraction was performed using the tuneRanger package^64^, which uses out of bag (OOB) mean squared error (MSQ) as an evaluation metric to find the best hyperparameters. Next, ten times repeated fourfold cross-validation was used to estimate out of sample accuracy. We report two accuracy measures: The Pearson correlation between the predicted scores and the actual scores was calculated using the test set and the trained model. Furthermore, we report the OOB, an out-of-sample accuracy measure provided by the RF algorithm. We repeated the above procedure (incl. hyperparameter tuning) on a dataset where the outcome variable was permuted (1000 permutations) to obtain a distribution of the accuracy measures under the null hypothesis. We used the median accuracy measure of the repeated CV procedure to obtain the *p*-value. Hyperparameter tuning was also performed in the permutation models to ensure that differences in accuracy measures are not due to overfitting the data. Finally, we corrected *p*-values for multiple testing using the Benjamini–Hochberg procedure^56^ as implemented in the package qvalue^65^. To extract biological meaning from the RF models, variable importances were calculated, including *p*-values using the method developed by Altmann et al.^61^ as implemented in ranger (1000 permutations). Note, that we compared the accuracy of RF models to other machine learning algorithms (LASSO and Elastic Net) using 10 times repeated fourfold crossvalidation (supplementary table 13) and only present the RF model in the main manuscript. Results of the LASSO model can be found in supplementary tables 13 and 14.

The performed analyses deviate somewhat from those that were preregistered on OSF (osf.io/wky4p). For example, sex and age are no longer modeled as a covariate because zBMI was used instead. Furthermore, model complexity was no longer reduced following the protocol by Bates et al.^66^. This is because the model had been simplified, and a maximal structure was reached without the need for model complexity reduction. Finally, through collaboration, we also were able to add exploratory random forest models to our analysis that complement our preregistered analyses.

## Ethics approval and consent to participate

The Faculty Ethics Committee of Radboud University approved the BIBO study (ECG 300107, ECG 22111/130112) in accordance with the Declaration of Helsinki. Mothers of the participants gave written informed consent before starting the study.

## Consent for publication

All authors have read and approved the manuscript as submitted.

## Results

### Descriptive statistics

**Figure 1:**
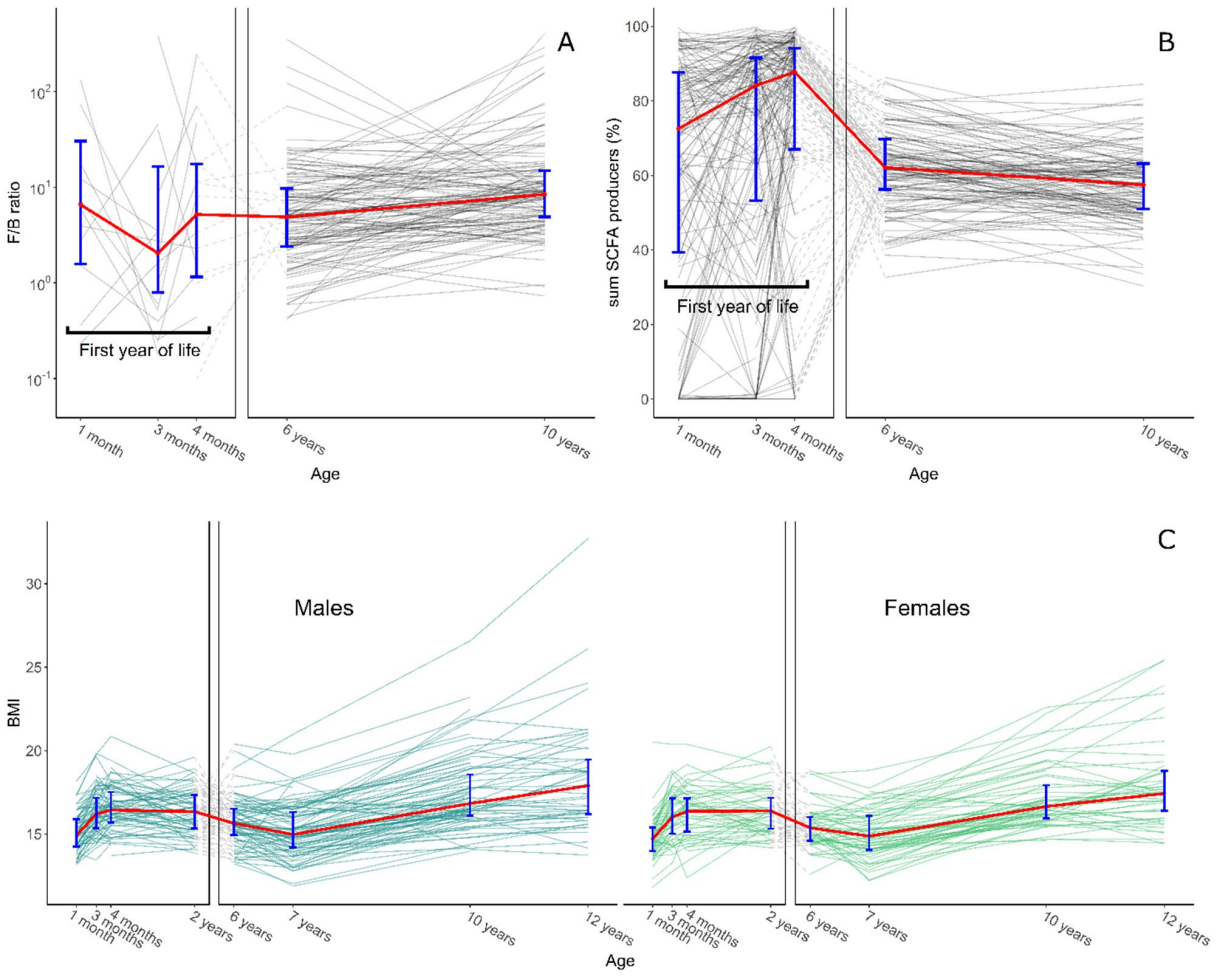
Individual and median trajectories of microbiota variables and BMI. (**A**) Individual and median (in red) trajectories of F/B ratio on log10 scale over time. (**B**) Individual and median trajectories of relative abundance of sum SCFA producers over time. (**C**) Individual and median (raw) BMI trajectories over time split by sex. Error bars indicate interquartile range (IQR). Please note the break in the x-axis. The lines crossing the break do not represent the actual slope and are only plotted to show individual growth trajectories.

**Figure 2:**
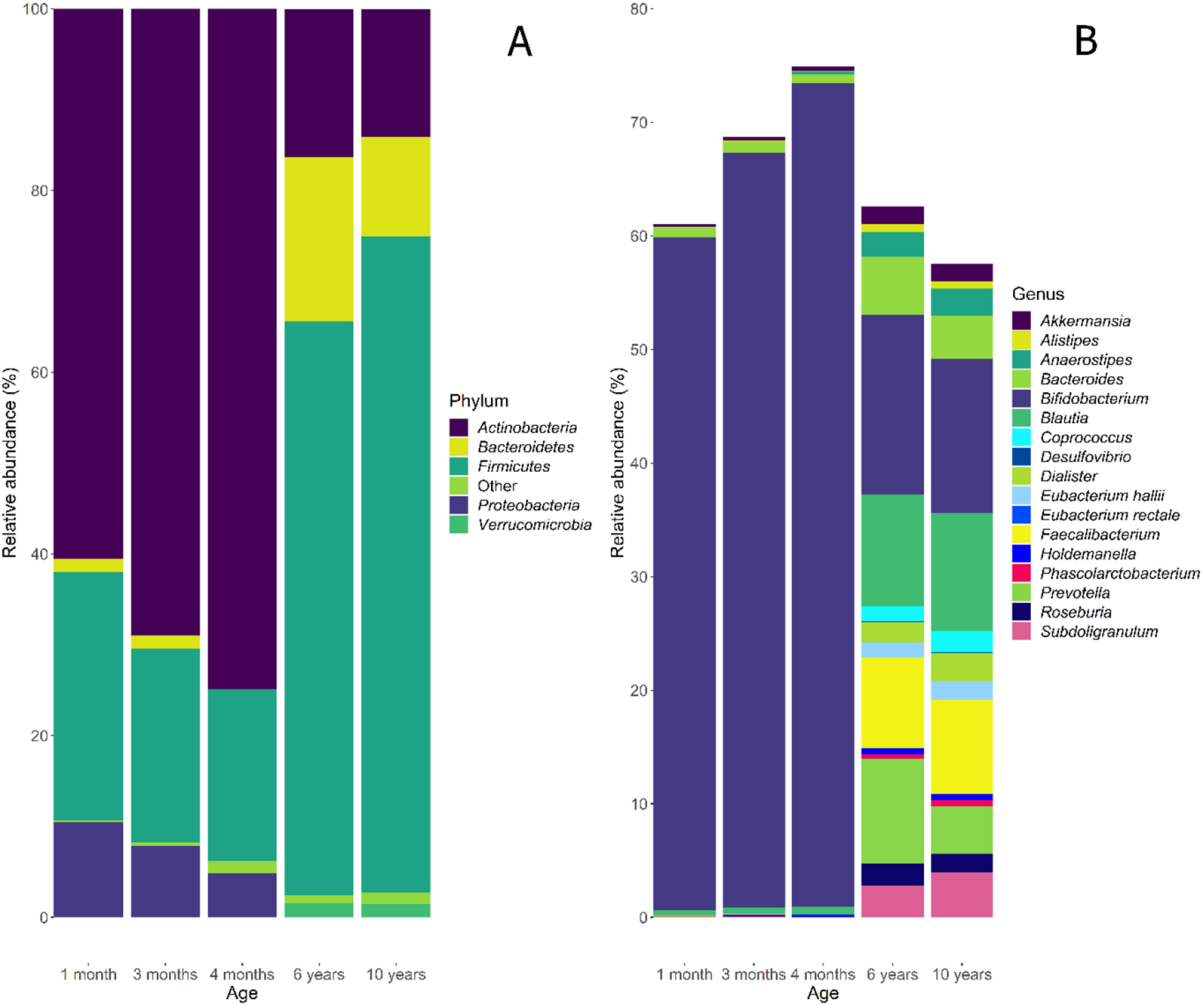
Gut microbiota composition over time. (**A**) The average relative abundance of the 6 most abundant phyla in the first 12 years of life are shown with Actinobacteria predominating in infancy and Firmicutes becoming the predominant phylum in childhood. Phyla with an abundance < 1% are plotted as “Other”. (**B**) The composition of average relative abundance of SCFA producer genera in the first 12 years of life is shown. In infancy, Bifidobacterium is the dominant genus of SCFA producers with the composition becoming more diverse in childhood. Note that y-axes are not equivalent.

The descriptive statistics per variable per time point can be seen in Table 3 and Fig. 1. There were missing values for different time points in both BMI and the microbiota data. For the correlation matrices, the data with missingness was used, and for the models, multiple imputation was applied. Because the phylum Bacteroidetes was not present in most of the samples in the first year of life, for many infants, the F/B ratio could not be calculated as the calculation resulted in an infinite value. Some of the calculated F/B ratios were in a higher range than what has been reported in the literature. In the current sample, the F/B ratio ranged between 0.06 and 482, which has not been reported before in the literature. These high values are due to the low relative abundance of phylum Bacteroidetes, which was at a relative abundance of less than 1% in most cases, especially in infants but also in older children. The overall composition of the gut microbiota at phylum level over time and the composition of the SCFA producer variable can be seen in Fig. 2. Birthweight (in grams) was available for 190 subjects (M = 3613, SD = 477).

**Table 3:** Descriptive statistics.

Variable	1 month		3 months		4 months		2 years		6 years		7 years		10 years		12 years	
	M (SD)	*n*	M (SD)	*n*	M (SD)	*n*	M (SD)	*n*	M (SD)	*n*	M (SD)	*n*	M (SD)	*n*	M (SD)	*n*
zBMI	−0.04 (0.92)	143	−0.36 (0.98)	148	−0.40 (0.95)	146	0.41 (0.93)	168	0.16 (0.88)	144	−0.29 (1.06)	157	0.23 (0.87)	156	−0.26 (1.13)	98^a^
F/B ratio	50.0 (91.1)	36	44.7 (115.5)	27	27.4 (60.9)	16			12.3 (34.9)	144			25.7 (60.5)	145		
Relative abundance Firmicutes	0.27 (0.26)	162	0.21 (0.25)	146	0.19 (0.25)	142			0.63 (0.13)	145			0.72 (0.10)	147		
Relative abundance Bacteroidetes	0.01 (0.05)	162	0.01 (0.04)	146	0.01 (0.06)	142			0.18 (0.15)	145			0.11 (0.09)	147		
Relative abundance SCFA producers	0.61 (0.33)	162	0.69 (0.32)	146	0.75 (0.29)	142			0.63 (0.11)	145			0.57 (0.09)	147		

Mean (M), standard deviation (SD) and sample size (*n*) are given per variable at each time point.

^a^The sample size for BMI was reduced at 12 years of age because measurement took place during a lab visit including an functional magnetic resonance imaging (fMRI) scan for which not all children met the inclusion criteria.

### Confirmatory analyses

**Figure 3:**
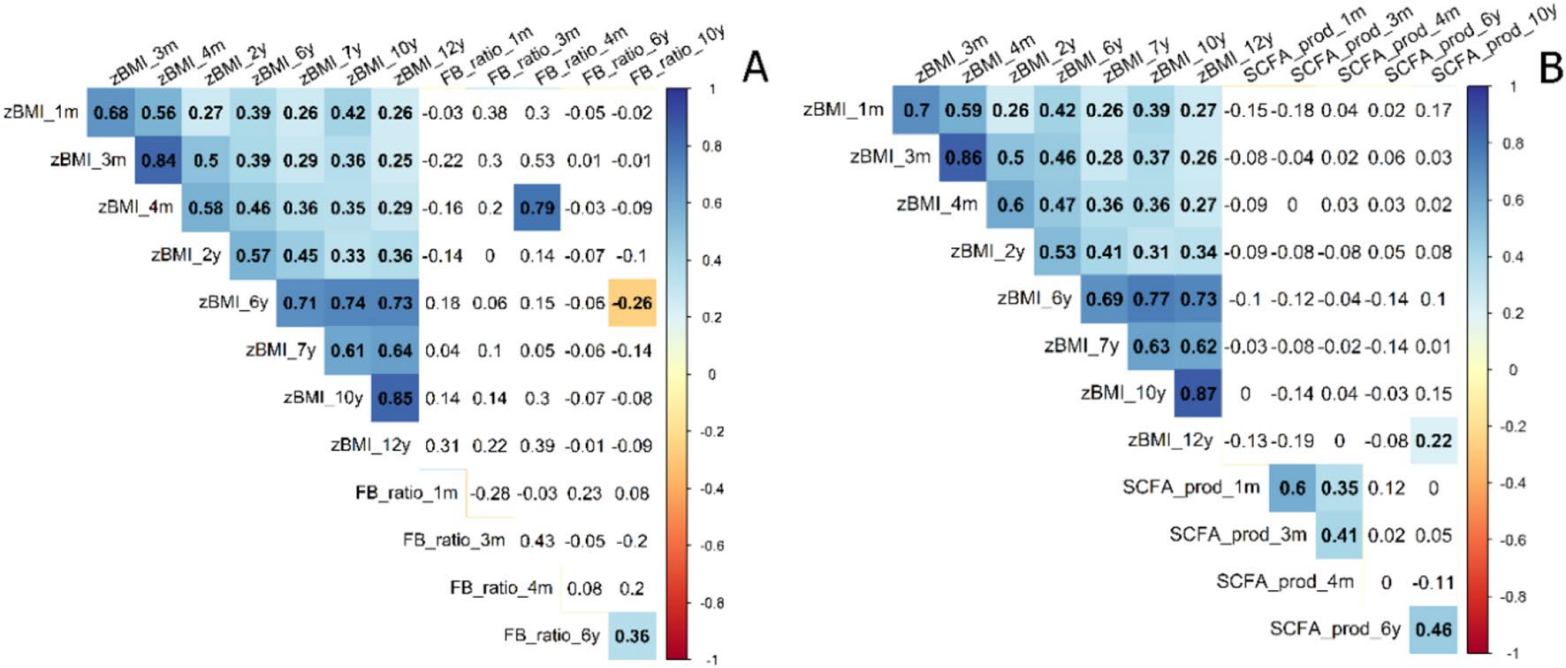
Spearman correlation matrices of main predictor and outcome varables. (**A**) Correlation Matrix between zBMI and F/B ratio over the ages. (**B**) Correlation matrix between zBMI and SCFA producers over the ages. Colored squares indicate significant values (*p* < 0.05).

We first investigated the relationship between zBMI and F/B ratio and SCFA producers at the same and subsequent time points by constructing two Spearman correlation matrices, including all time points of the relevant variables. The first correlation matrix (Fig. 3A) showed two significant associations between F/B ratio and zBMI. These relations were between F/B ratio and zBMI at 4 months (r = 0.79, *p* = 0.004, *n* = 11) and between F/B ratio at 10 years and zBMI at 6 years (r = −0.26, *p* = 0.003, *n* = 126). The same correlation matrix was constructed for associations between zBMI and SCFA producers (Fig. 3B). In this matrix, only SCFA producers at 10 years was significantly associated with zBMI at 12 years (r = 0.22, *p* = 0.047, *n* = 89). After applying the Benjamini–Hochberg procedure to the hypothesized effects, none of these relationships remained significant (FDR < 0.05).

To investigate whether F/B ratio and SCFA producers could predict BMI over time, multiple Bayesian linear multilevel models were fitted. The models were constructed with the outcome variable zBMI at 3 and 4 months, 2, 7, and 12 years while allowing the intercept to vary between individuals. The model predictors were F/B ratio or the sum score of SCFA producers (see “Methods”), respectively, that were modeled as fixed and random effect. For all models, the covariate structure was identical: We included birthweight and zBMI of the previous time point (T−1) at ages 1, 3, 4 months, and 6 and 10 years. The following formula shows the model structure at the example of F/B ratio:

$$ {\text{zBMI}}\sim {\text{F}}/{\text{B}}\;{\text{ratio}}\left( {T - 1} \right) + {\text{zBMI}}\left( {T - 1} \right) + {\text{birthweight}} + \left( {1 + {\text{F}}/{\text{B}}\;{\text{ratio}}\left( {T - 1} \right)|{\text{subject}}} \right) $$

Tables with all parameter estimates for all models can be found in supplementary Tables 1–10. In the following, we describe the main parameters of interest regarding our research questions.

#### F/B ratio

Because the F/B ratio could not be calculated in infancy, we fitted the linear model only to the childhood samples and found no association between F/B ratio and zBMI ($\upbeta$ = 0.069, 95% CI [−0.267, 0.437]). We explored the relationship between Firmicutes, Bacteroidetes, and zBMI further by entering the clr-vectors of both Firmicutes and Bacteroidetes as independent predictors into one model. This allowed us to explore potential relationships between Bacteroidetes and Firmicutes also in infancy. The model showed that relative abundance of Firmicutes (β = −0.078, 95% CI [−0.136, -0.018]) and Bacteroidetes (β = −0.060, 95% CI [−0.096, −0.027]) were both negatively associated with future BMI. This relationship remained when examining only infant samples (Firmicutes: β = −0.039, 95% CI [−0.088, 0.007], Bacteroidetes: β = −0.047, 95% CI [−0.090, −0.014]) and showed even stronger associations in childhood (Firmicutes: β = −0.263, 95% CI [−0.431, −0.092], Bacteroidetes: β = −0.161, 95% CI [−0.239, −0.068]).

#### SCFA producers

**Figure 4:**
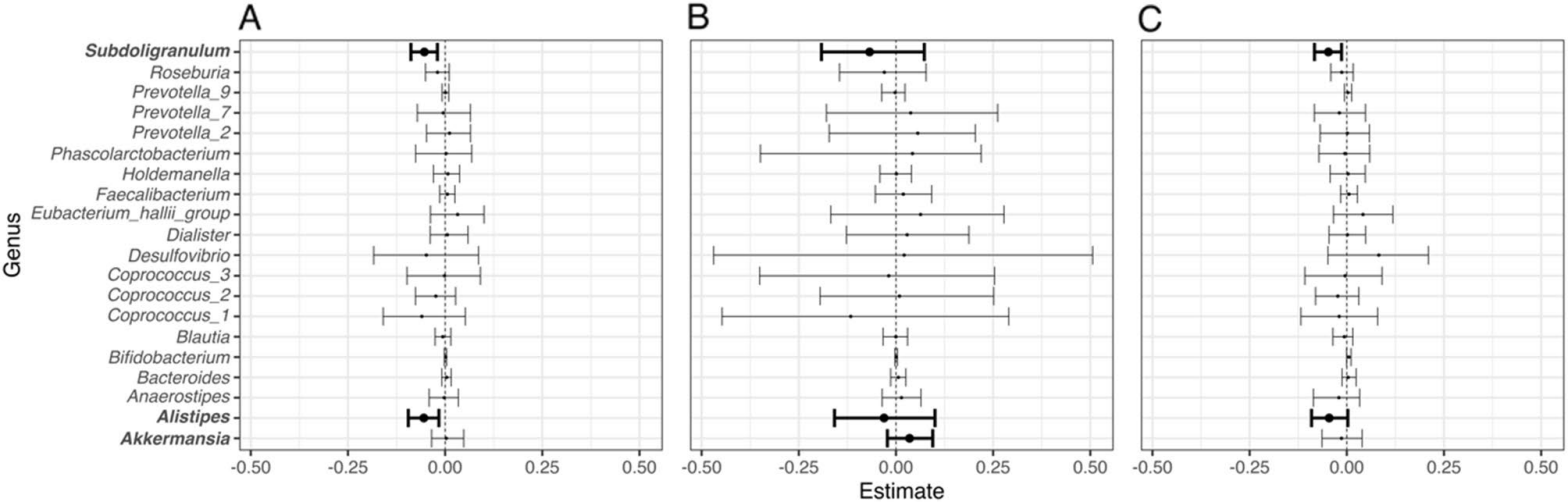
Posterior distribution (median and 95% credible interval) of $\upbeta$ coefficients (slopes) per SCFA producer. (**A**) All samples. (**B**) Only infant samples. (**C**) Only childhood samples.

SCFA producers (sum score) were not associated with future zBMI ($\upbeta$= 0.005, 95% CI [−0.033, 0.044]. We then explored the same model with SCFA producers as independent predictors rather than summed to obtain a better insight into how single SCFA producers relate to zBMI. Figure 4A summarizes the posterior distributions of the slope parameters (median including 95% credible interval) for each genus: The model was uncertain about most slope estimates while it confidently indicated that Subdoligranulum and Alistipes were negatively associated with future zBMI. To investigate whether these relationships remained in either infancy or childhood independently, we fitted the same model using either only infant samples (Fig. 4B) or childhood samples (Fig. 4C) and observe similar trends. The model using infant samples was less certain about the slope parameters of Subdoligranulum and Alistipes. Here, most of the posterior distribution of the parameter reflecting Akkermansia being positive (> 88%) might indicate a potential small positive association with future zBMI early in life. In general, credible intervals were wider in the infant model compared to the childhood model reflecting higher uncertainty of the model about the parameter estimates of these slopes. This can be explained by the predominance of Bifidobacterium and lower prevalence of other genera in infancy.

### Exploratory analyses

#### Random forests models

We fitted two RF models for each time point after hyperparameter tuning (see supplementary table 11 for hyperparameters): One model predicting the future zBMI and one cross-sectional model. Table 4 shows the accuracy measures, including corresponding *p*-values and false discovery rates (FDR) for each RF model. If there was a relationship between microbial composition and zBMI, we would expect a significantly higher correlation and lower out of bag error (OOB) for the RF models that used the non-permuted outcome compared to the null-model that used the permuted outcome (1000 permutations). Three models reached a *p*-value < 0.05 for either OOB or Pearson. Neither of those remained significant when restricting to FDR < 0.1.

**Table 4:** Accuracy of the Random Forest models.

Time microbiota	Time zBMI	Accuracy measure	Median	*P*-value	Q-value
1 m	1 m	OOB	0.850	0.363	0.667
1 m	1 m	Pearson	0.000	0.544	0.667
1 m	3 m	OOB	0.937	0.477	0.667
1 m	3 m	Pearson	0.018	0.540	0.667
3 m	3 m	OOB	0.967	0.780	0.821
3 m	3 m	Pearson	−0.100	0.775	0.821
3 m	4 m	OOB	0.897	0.889	0.889
3 m	4 m	Pearson	0.135	0.228	0.570
**4 m**	**4 m**	**OOB**	**0.857**	**0.019**	**0.190**
**4 m**	**4 m**	**Pearson**	**0.249**	**0.053**	**0.212**
4 m	2y	OOB	0.950	0.315	0.667
4 m	2y	Pearson	0.160	0.183	0.523
6y	6y	OOB	0.790	0.554	0.667
6y	6y	Pearson	0.047	0.432	0.667
6y	7y	OOB	1.140	0.567	0.667
6y	7y	Pearson	0.011	0.533	0.667
**10y**	**10y**	**OOB**	**0.783**	**0.044**	**0.212**
**10y**	**10y**	**Pearson**	**0.258**	**0.088**	**0.293**
**10y**	**12y**	**OOB**	**1.131**	**0.019**	**0.190**
**10y**	**12y**	**Pearson**	**0.322**	**0.032**	**0.212**

OOB = Out of Bag error of the Random Forest models. Median = Median accuracy score (either OOB or Pearson) from the 10 times repeated fourfold crossvalidation procedure. Models that reached *p* < .05 for either Pearson or OOB are printed in bold.

Nevertheless, we computed permutation variable importances including their *p*-values for the three models with *p* < 0.05 that had an FDR <  = 0.21 for exploratory purposes according to the method of Altmann et al.^61^. The variable importance values reflect the mean decrease in accuracy when the corresponding predictor is permuted. Above mentioned three models are listed as model 1, model 2 and model 3 and can be seen in supplementary table 12 where we show the top 10 important features (genera) per significant model. In light of our research question, we indicated whether any of the top 10 genera is included in our list of SCFA producers. For the cross-sectional model at 4 months of age (model 1), Akkermansia as a SCFA producer is of the highest importance, followed by genera belonging to the Firmicutes and Actinobacteria. The cross-sectional model at 10 years (model 2) includes mostly Bacteroidetes and Firmicutes as features of high importance, among which the SCFA producing genera Bacteroides, Roseburia, and Prevotella_9. Finally, the 10 year model predicting 12 years zBMI (model 3) only indicated Ruminococcus_2 and Parabacteroides, which belong to the phylum Firmicutes and Bacteroidetes, respectively, among the significantly important genera.

## Discussion

Our results indicate that there is no decisive evidence to support the energy harvesting hypothesis for the relation between gut microbiota and obesity in infancy and childhood. Contrary to expectation, we only found few statistically significant associations between zBMI and F/B ratio or SCFA producers, which were no longer significant after the application of the Benjamini–Hochberg procedure. Bayesian multilevel models also did not show associations between future zBMI and F/B ratio or SCFA producers. However, exploratory analyses revealed that the relative abundances of Firmicutes and Bacteroidetes were independently negatively associated with zBMI between infancy and 12 years of age after adjusting for previous time point zBMI and birthweight. Further exploration of SCFA producers showed that genera Subdoligranulum and Alistipes were negatively related to future zBMI in childhood but not in infancy. Our RF models also suggest that multiple SCFA producers could be associated with zBMI at different time points.

The lack of association between F/B ratio and BMI is in line with previous research which suggest no differences in F/B ratio between lean and obese individuals^22–24,67^. Our findings are in agreement with one study in children that did not find evidence to support the F/B ratio as a biomarker of obesity^30^ but contrary to two other studies in children^28,68^. Our observations can possibly be explained by our study's low number of obese children. Only 28 out of 1160 BMI observations reached a zBMI > 2, which corresponds with obesity according to the WHO guidelines^43^. Most of the previous research on gut microbiota and BMI has compared obese and lean individuals in groups, whereas we modeled BMI as a continuous variable. It is possible that the relationship between F/B ratio and BMI only exists when BMI is more than two standard deviations away from the mean. For example, in one study with Kazakh children^29^, the F/B ratio association was only found in children that are considered obese. Related to our results, this could suggest that for a normal BMI in a healthy population, F/B ratio is not associated with increased BMI and that the F/B ratio is only related to more extreme values of BMI. However, early evidence supporting the energy harvesting hypothesis would suggest that gut microbiota changes should gradually occur as BMI increases^13,14^. One study in adults also suggested that F/B ratio may be more strongly associated with metabolic markers correlated with obesity like insulin resistance, non-alcoholic fatty liver disease and metabolic syndrome rather than obesity^69^, all of which would be rare in our pediatric sample. Overall, our results suggest that the F/B ratio may not be a valid universal biomarker of obesity in children, especially during infancy.

Our exploratory analyses show that the overall relative abundances of Firmicutes and Bacteroidetes were negatively associated with future BMI, which is in line with the meta-analysis by Kim et al.^32^. They found that Firmicutes and Bacteroidetes were independently and negatively associated with BMI between 11 and 47, albeit not statistically significantly. Our findings could also be related to previous research that found that the gut microbiota's overall richness is negatively associated with obesity^17,70^. Indeed, post hoc analyses of our sample showed that observed richness (number of taxa per sample) was significantly negatively associated with BMI. These findings suggest an overall negative relationship between Firmicutes, Bacteroidetes, microbial richness, and BMI in the first 13 years of life. In part, this relationship may be explained by differences in dietary patterns, which are a significant factor in both weight gain and the diversity of the microbiota^6^. For example, probiotics, prebiotics, and dietary fiber have been associated with improvements in body composition and increases of several specific bacterial species^6,71^.

There are several differences between our study and previous research that should be mentioned. Most importantly, the sample sizes of F/B ratios in the infancy samples were small due to the high number of zero counts of Bacteroidetes in infants, and the range was extremely large compared to previous research. This large range can be attributed to the low abundance of Bacteroidetes (< 1%) in some of the samples, especially during infancy, which resulted in a high F/B ratio. To our knowledge, the only other study reporting the F/B ratio in infancy is that by Mariat and colleagues^51^, who calculated the F/B ratio for 21 infants between the ages of 3 weeks and 10 months of age. They found F/B ratios between 0 and 23, whereas we found F/B ratios between 0 and 482 at 1, 3, and 4 months of age. Our high values need not be outside the realm of possibilities because the infant gut microbiota is mostly dominated by Bifidobacterium and is increasingly colonized with Bacteroidetes over time^32,51^. This pattern is also observed in the current sample as the relative abundance of Bacteroidetes at ages 6 and 10 increased compared to the first year of life.

A reason for the low abundance of Bacteroidetes could be the geographical location. One study with 606 infants of 6 weeks of age across Europe found that the genus Bacteroides, which belongs to the Bacteroidetes phylum, was reduced in Northern European infants compared to more Southern European infants^72^. Similarly, the gut microbiota of 1 to 6 year old children in Burkina Faso was significantly enriched in Bacteroidetes compared to that of Italian children^73,74^. This finding suggests that the current sample, which consisted of Dutch children, may have fewer Bacteroidetes than other samples, such as the study by Mariat et al.^51^, conducted in France. Differences in gut composition between geographical locations can, in turn, be due to cultural differences in lifestyle and dietary patterns^75^.

Similar to the F/B ratio, no significant associations were found between the aggregated SCFA producers variable and BMI. This is contrary to a large body of literature, which suggests a positive relationship between obesity and SCFAs, but in line with literature showing no or an inverse association between obesity and SCFAs^33–39^. For example, a recent meta-analysis found that elevated levels of SCFA in stool and plasma were associated with obesity in individuals aged 6 to 74^32^. Because the current study did not assess SCFA concentrations in stool or plasma, we could not directly replicate those findings. However, in theory, our novel SCFA producer variable representing the sum of relative abundances of carefully selected SCFA producer genera should be correlated to SCFA concentrations in stool and plasma. This assumption awaits further experimental validation in future studies. Furthermore, in exploratory analyses consisting of multilevel and RF models, we found evidence that specific SCFA producer genera are associated with BMI. Out of the six genera (Subdoligranulum, Alistipes, Akkermansia, Bacteroides, Prevotella, and Roseburia) that were detected, all but Subdoligranulum have been identified as propionate producers (Table 2).

Although we cannot gauge the direction of the associations of the genera that were detected with the RF algorithm, the two genera detected in the multilevel models, Alistipes and Subdoligranulum, were negatively associated with BMI during childhood. One previous study found Alistipes to be negatively associated with obesity in German adults^76^. Another study found that in cases where Alistipes was more abundant at baseline in participants of a weight loss invervention, those participants were more succesful in lowering and maintaining weight compared to individuals lower in Alistipes abundance at baseline^69^. The genus Subdoligranulum has also been positively associated with overall metabolic health, including lower BMI and fat mass in human adults^77^. However, further investigation of this relationship, through the cultivation of Subdoligranulum in obese mice, did not result in differences in body weight and fat mass^77^. Our results are thus in line with previous studies that found a negative association between Alistipes, Subdoligranulum and obesity, but further studies are needed to investigate potential causal mechanisms.

The SCFAs produced by Alistipes and Subdoligranulum, butyrate and propionate, are both reported to be associated with reduced obesity in previous research through different mehcanisms^36,38,39,78^. For example, supplementation of butyrate seemed to prevent diet-induced obesity in mice through reduced appetite^33,79,80^. Propionate has also received increased attention due to its involvement in appetite regulation of glucose metabolism^81,82^. Rodent and adult human studies have shown that the supplementation of propionate led to reductions in food intake, subjective appetite, body weight, and insulin sensitivity improvement^38,39^. Chambers et al.^39^ found that the supplementation of an inulin-propionate ester (IPE) or inulin both led to reductions of individual propionate producers (Blautia and Roseburia) but also to increases of others (Bacteroidetes). In addition to inulin, supplementation of other dietary fibers like arabinoxylan in obese and overweight individuals led to an increased fecal propionate concentration and increases of Blautia obeum and Prevotella copri, both known propionate producers^83^. Our exploratory study results are thus in line with findings in rodents and human adults, which suggest a potential anti-obesity effect of propionate and butyrate, that may already be present in the first 12 years of life. It is still curious that Chambers et al.^39^ also found that certain propionate-producing species were reduced. This observation could be explained by dosage effects^39^, but possibly also by cross-feeding and bacterial interaction effects, which are known to play a role in SCFA production^84^. Overall, our findings indicate that not the overall sum of SCFA producers is related to BMI, but rather specific SCFA producer genera.

Strengths of our study are the use of an innovative theory-based biomarker, sophisticated statistics, and pre-registration for scientific transparency purposes. Additional strengths are its prospective longitudinal nature, healthy population and length of the assessment period, covering a relevant period in the development of obesity, namely from infancy to puberty. A limitation of our study and possible explanation for why we did not find evidence supporting the energy harvesting hypothesis is that many bacterial species may still be unidentified, or their functional role still unknown^21,85^. The bacterial genera used in creating the SCFA producers variable is not necessarily an exhaustive list, and more could still be identified. Until future research informs a more comprehensive overview of bacterial species and their activity, it may be hard to capture the 'true' SCFA producers. Furthermore, the production of SCFAs also depends on the nutritional intake, as SCFA production requires the fermentation of non-digestible carbohydrates^86^. Thus, the availability of resources and the interaction between different bacteria make it more challenging to determine the production of SCFAs.

Future studies that investigate (the development of) obesity and BMI should focus on analyzing SCFA excretion and individual SCFA producers in relation to other associated lifestyle factors. For example, one could measure SCFAs in both serum and stool and compare those values with the known SCFA producers as listed in the methods. Studies could also focus on dietary intake and interaction effects as they play an essential role in the gut composition and SCFA production. By gaining more insight into the complexity of the gut bacteria, their metabolism, and their interactions with the host, it may be possible in the future to construct a sensitive biomarker for the early detection of obesity. Furthermore, the microbiome research field is still in development and innovating in its methods. Many studies used in the theoretical framework for this study differ in their approaches. For example, studies often report on different taxonomic levels (e.g., phylum vs. genus). There also exist differences between studies due to the wide variety of methodology and pipelines used to detect and analyze microorganisms^17^ and data transformation methods^55^. Differential analytical approaches do lead to diverging conclusions, making it harder to compare studies with each other. Hypothesis testing of previous findings and theories in a replicable way will be essential to make progress in the microbiome field.

## Conclusion

Based on the current findings, there is little evidence to support a link between the F/B ratio and BMI, and the sum of known SCFA producers and BMI, in the first 12 years of life. Especially during infancy, the F/B ratio may be inadequate because Bacteroidetes are not present in large enough proportions. The lack of Bacteroidetes in the sample during infancy could be explained by geographic location. However, we also found no association between F/B and zBMI at ages 6 and 10, showing that our results are not entirely due to the lack of Bacteroidetes in infancy. We also did not find the hypothesized relationship between the sum of SCFA producers and zBMI. Exploratory analyses showed that Firmicutes and Bacteroidetes were individually negatively related to zBMI changes throughout the first 12 years of life, and that the SCFA producing genera Subdoligranulum (butyrate) and Alistipes (propionate) were negatively related to future zBMI in childhood. This last finding is in line with the literature on propionate supplementation, which suggests an anti-obesity effect of propionate. Given our results and the literature's heterogeneity, it is not recommended to use the F/B ratio or SCFA producers as a universal predictor biomarker for BMI in childhood, especially in infants. Future studies should investigate individual SCFA producing genera specifically and consider other factors such as cross-feeding and microbial interactions in addition to diet and lifestyle in studying the gut microbiota in BMI development.

## Data availability

The data that support the findings of this study are available upon reasonable request. The data are not freely available, as we did not ask participants to consent to store de-identified data in an online depository. Deidentified individual participant data that underlie the results reported in this article will be made available upon publication (no end date) to researchers with a methodologically sound proposal for re-use of the data. The proposal should be directed to carolina.deweerth@radboudumc.nl. Upon approval, data requestors will need to sign a data transfer agreement. Researchers are asked to analyze the data and/or publish the results within two years. The scripts used for the analyses can be found here: https://doi.org/10.5281/zenodo.5268995.

## Supplementary Information

Supplementary Information.
