# Supplementary material for: Gut microbiota and BMI throughout childhood: the role of firmicutes, bacteroidetes, and short-chain fatty acid producers
Source: Sci Rep. 2022 Feb 24;12:3140. doi: 10.1038/s41598-022-07176-6 (PMC8873392; doi:10.1038/s41598-022-07176-6)
Supplement: Supplementary file 1 — Supplementary Information. [file 41598_2022_7176_MOESM1_ESM.pdf]

Supplementary Tables

Table 1: Coefficients for the model using child samples and formula: bmi ~ bmi__tminus + s__fb__ratio + s__bw__grams + (1 + s__fb__ratio | id)

Parameter	Estimate	SD	95% CI
b__Intercept	-0.46	0.10	-0.66 : -0.24
b__bmi__tminus	0.88	0.06	0.77 : 1.00
b__s__bw__grams	-0.01	0.05	-0.12 : 0.10
b__s__fb__ratio	0.07	0.17	-0.27 : 0.44
cor__id___Intercept___s__fb__ratio	0.08	0.58	-0.94 : 0.96
sd__id___Intercept	0.13	0.11	0.00 : 0.38
sd__id___s__fb__ratio	0.15	0.18	0.01 : 0.64
sigma	0.70	0.04	0.61 : 0.77

Table 2: Coefficients for the model using all samples and formula: bmi ~ bmi__tminus + Bacteroidetes + Firmicutes + s__bw__grams + (1 + Bacteroidetes + Firmicutes | id)

Parameter	Estimate	SD	95% CI
b__Bacteroidetes	-0.06	0.02	-0.10 : -0.03
b__Firmicutes	-0.08	0.03	-0.14 : -0.02
b__Intercept	0.33	0.14	0.07 : 0.62
b__bmi__tminus	0.72	0.03	0.66 : 0.79
b__s__bw__grams	-0.01	0.03	-0.07 : 0.06
cor__id___Bacteroidetes___Firmicutes	-0.01	0.49	-0.86 : 0.87
cor__id___Intercept___Bacteroidetes	0.02	0.49	-0.87 : 0.87
cor__id___Intercept___Firmicutes	-0.34	0.52	-0.95 : 0.81
sd__id___Bacteroidetes	0.03	0.02	0.00 : 0.07
sd__id___Firmicutes	0.02	0.02	0.00 : 0.06
sd__id___Intercept	0.08	0.07	0.00 : 0.29
sigma	0.80	0.03	0.75 : 0.85

Table 3: Coefficients for the model using infant samples and formula: bmi ~ bmi__tminus + Bacteroidetes + Firmicutes + s__bw__grams + (1 + Bacteroidetes + Firmicutes | id)

Parameter	Estimate	SD	95% CI
b__Bacteroidetes	-0.05	0.02	-0.09 : -0.01
b__Firmicutes	-0.04	0.03	-0.09 : 0.01
b__Intercept	0.16	0.12	-0.08 : 0.39
b__bmi__tminus	0.69	0.04	0.61 : 0.76

Parameter	Estimate	SD	95% CI
b_s_bw_grams	0.00	0.04	-0.07 : 0.07
cor_id___Bacteroidetes___Firmicutes	-0.04	0.50	-0.90 : 0.87
cor_id___Intercept___Bacteroidetes	-0.01	0.50	-0.89 : 0.88
cor_id___Intercept___Firmicutes	-0.15	0.50	-0.91 : 0.83
sd_id___Bacteroidetes	0.02	0.02	0.00 : 0.07
sd_id___Firmicutes	0.01	0.01	0.00 : 0.04
sd_id___Intercept	0.03	0.04	0.00 : 0.13
sigma	0.79	0.02	0.75 : 0.84

Table 4: Coefficients for the model using child samples and formula: bmi ~ bmi_tminus + Bacteroidetes + Firmicutes + s_bw_grams + (1 + Bacteroidetes + Firmicutes | id)

Parameter	Estimate	SD	95% CI
b_Bacteroidetes	-0.16	0.04	-0.24 : -0.07
b_Firmicutes	-0.26	0.09	-0.43 : -0.09
b_Intercept	1.64	0.50	0.67 : 2.65
b_bmi_tminus	0.88	0.06	0.77 : 1.00
b_s_bw_grams	-0.11	0.06	-0.22 : -0.01
cor_id___Bacteroidetes___Firmicutes	-0.25	0.51	-0.93 : 0.82
cor_id___Intercept___Bacteroidetes	-0.03	0.50	-0.89 : 0.85
cor_id___Intercept___Firmicutes	0.03	0.47	-0.87 : 0.88
sd_id___Bacteroidetes	0.03	0.04	0.00 : 0.16
sd_id___Firmicutes	0.05	0.04	0.00 : 0.12
sd_id___Intercept	0.05	0.07	0.00 : 0.24
sigma	0.67	0.09	0.47 : 0.80

Table 5: Coefficients for the model using all samples and formula: bmi ~ bmi_tminus + scfa_clr + s_bw_grams + (1 + scfa_clr | id)

Parameter	Estimate	SD	95% CI
b_Intercept	-0.25	0.13	-0.51 : 0.02
b_bmi_tminus	0.67	0.04	0.60 : 0.74
b_s_bw_grams	0.03	0.03	-0.04 : 0.10
b_scfa_clr	0.01	0.02	-0.03 : 0.04
cor_id___Intercept___scfa_clr	-0.66	0.57	-0.99 : 0.87
sd_id___Intercept	0.09	0.11	0.00 : 0.41
sd_id___scfa_clr	0.01	0.01	0.00 : 0.06
sigma	0.84	0.02	0.80 : 0.89

Table 6: Coefficients for the model using infant samples and formula: bmi ~ bmi_tminus + scfa_clr + s_bw_grams + (1 + scfa_clr | id)

Parameter	Estimate	SD	95% CI
b_Intercept	-0.07	0.13	-0.30 : 0.18
b_bmi_tminus	0.67	0.04	0.58 : 0.75
b_s_bw_grams	0.02	0.04	-0.06 : 0.11
b_scfa_clr	0.01	0.02	-0.03 : 0.05
cor_id__Intercept__scfa_clr	-0.66	0.56	-1.00 : 0.86
sd_id__Intercept	0.10	0.14	0.00 : 0.49
sd_id__scfa_clr	0.01	0.02	0.00 : 0.06
sigma	0.80	0.03	0.75 : 0.86

Table 7: Coefficients for the model using child samples and formula: bmi ~ bmi_tminus + scfa_clr + s_bw_grams + (1 + scfa_clr | id)

Parameter	Estimate	SD	95% CI
b_Intercept	-0.15	0.47	-1.09 : 0.78
b_bmi_tminus	0.79	0.05	0.70 : 0.89
b_s_bw_grams	-0.03	0.04	-0.12 : 0.06
b_scfa_clr	-0.01	0.07	-0.17 : 0.11
cor_id__Intercept__scfa_clr	-0.38	0.58	-0.99 : 0.89
sd_id__Intercept	0.23	0.19	0.01 : 0.73
sd_id__scfa_clr	0.03	0.03	0.00 : 0.10
sigma	0.76	0.04	0.69 : 0.84

Table 8: Coefficients for the model using all_samples and formula: bmi ~ bmi_tminus + Akkermansia + Alistipes + Anaerostipes + Bacteroides + Bifidobacterium + Blautia + Coprococcus_1 + Coprococcus_2 + Coprococcus_3 + Desulfovibrio + Dialister + Faecalibacterium + Holdemanella + Phascolarctobacterium + Prevotella_2 + Prevotella_7 + Prevotella_9 + Roseburia + Subdoligranulum + s_bw_grams + Eubacterium_hallii_group + (1 | id)

Parameter	Estimate	SD	95% CI
b_Akkermansia	0.01	0.02	-0.02 : 0.05
b_Alistipes	-0.05	0.02	-0.09 : -0.02
b_Anaerostipes	0.00	0.02	-0.04 : 0.03
b_Bacteroides	0.00	0.01	-0.01 : 0.01
b_Bifidobacterium	0.00	0.00	0.00 : 0.00
b_Blautia	-0.01	0.01	-0.02 : 0.01
b_Coprococcus_1	-0.04	0.04	-0.12 : 0.04
b_Coprococcus_2	0.02	0.02	-0.03 : 0.07
b_Coprococcus_3	0.05	0.04	-0.03 : 0.13
b_Desulfovibrio	-0.05	0.07	-0.18 : 0.09
b_Dialister	0.02	0.02	-0.02 : 0.06
b_Eubacterium_hallii_group	0.03	0.03	-0.03 : 0.09

Parameter	Estimate	SD	95% CI
b__Faecalibacterium	0.00	0.01	-0.02 : 0.02
b__Holdemanella	0.01	0.01	-0.01 : 0.04
b__Intercept	0.00	0.05	-0.10 : 0.10
b__Phascolarctobacterium	0.01	0.03	-0.05 : 0.06
b__Prevotella__2	0.02	0.02	-0.03 : 0.07
b__Prevotella__7	-0.01	0.02	-0.06 : 0.02
b__Prevotella__9	0.00	0.00	-0.01 : 0.01
b__Roseburia	-0.04	0.01	-0.06 : -0.01
b__Subdoligranulum	-0.04	0.02	-0.07 : -0.01
b__bmi__tminus	0.73	0.03	0.67 : 0.78
b__s__bw__grams	-0.02	0.03	-0.07 : 0.04
sd__id___Intercept	0.06	0.04	0.00 : 0.17
sigma	0.79	0.02	0.75 : 0.83

Table 9: Coefficients for the model using infant samples and formula: bmi ~ bmi__tminus + Akkermansia + Alistipes + Anaerostipes + Bacteroides + Bifidobacterium + Blautia + Coprococcus__1 + Coprococcus__2 + Coprococcus__3 + Desulfovibrio + Dialister + Faecalibacterium + Holdemanella + Phascolarctobacterium + Prevotella__2 + Prevotella__7 + Prevotella__9 + Roseburia + Subdoligranulum + s__bw__grams + Eubacterium__hallii__group + (1 | id)

Parameter	Estimate	SD	95% CI
b__Akkermansia	0.04	0.03	-0.01 : 0.09
b__Alistipes	0.02	0.05	-0.08 : 0.12
b__Anaerostipes	0.01	0.03	-0.04 : 0.06
b__Bacteroides	0.01	0.01	-0.01 : 0.02
b__Bifidobacterium	0.00	0.00	0.00 : 0.00
b__Blautia	-0.01	0.01	-0.03 : 0.02
b__Coprococcus__1	-0.11	0.12	-0.33 : 0.13
b__Coprococcus__2	0.08	0.10	-0.13 : 0.28
b__Coprococcus__3	-0.04	0.11	-0.26 : 0.18
b__Desulfovibrio	0.02	0.25	-0.47 : 0.51
b__Dialister	-0.02	0.07	-0.15 : 0.11
b__Eubacterium__hallii__group	-0.07	0.10	-0.27 : 0.14
b__Faecalibacterium	0.03	0.05	-0.06 : 0.12
b__Holdemanella	0.01	0.02	-0.03 : 0.05
b__Intercept	0.00	0.09	-0.18 : 0.17
b__Phascolarctobacterium	-0.04	0.08	-0.18 : 0.11
b__Prevotella__2	0.14	0.07	-0.01 : 0.28
b__Prevotella__7	-0.03	0.05	-0.13 : 0.06
b__Prevotella__9	0.01	0.01	-0.02 : 0.03
b__Roseburia	-0.03	0.05	-0.13 : 0.07
b__Subdoligranulum	-0.06	0.06	-0.17 : 0.04
b__bmi__tminus	0.65	0.04	0.56 : 0.71
b__s__bw__grams	0.00	0.04	-0.06 : 0.08
sd__id___Intercept	0.05	0.05	0.00 : 0.17
sigma	0.80	0.02	0.75 : 0.85

Table 10: Coefficients for the model using child samples and formula: bmi ~ bmi_tminus + Akkermansia + Alistipes + Anaerostipes + Bacteroides + Bifidobacterium + Blautia + Coprococcus_1 + Coprococcus_2 + Coprococcus_3 + Desulfovibrio + Dialister + Faecalibacterium + Holdemanella + Phascolarctobacterium + Prevotella_2 + Prevotella_7 + Prevotella_9 + Roseburia + Subdoligranulum + s_bw_grams + Eubacterium_hallii_group + (1 | id)

Parameter	Estimate	SD	95% CI
b_Akkermansia	-0.02	0.02	-0.06 : 0.03
b_Alistipes	-0.07	0.02	-0.10 : -0.03
b_Anaerostipes	-0.06	0.03	-0.12 : 0.00
b_Bacteroides	0.00	0.01	-0.02 : 0.01
b_Bifidobacterium	0.00	0.00	-0.01 : 0.01
b_Blautia	-0.01	0.01	-0.03 : 0.00
b_Coprococcus_1	-0.04	0.05	-0.13 : 0.05
b_Coprococcus_2	0.01	0.03	-0.04 : 0.06
b_Coprococcus_3	0.00	0.04	-0.08 : 0.09
b_Desulfovibrio	0.08	0.07	-0.05 : 0.21
b_Dialister	0.03	0.02	-0.01 : 0.06
b_Eubacterium_hallii_group	0.02	0.03	-0.04 : 0.08
b_Faecalibacterium	-0.01	0.01	-0.03 : 0.01
b_Holdemanella	0.01	0.02	-0.03 : 0.04
b_Intercept	0.49	0.15	0.19 : 0.78
b_Phascolarctobacterium	0.00	0.03	-0.05 : 0.05
b_Prevotella_2	0.00	0.02	-0.05 : 0.05
b_Prevotella_7	-0.03	0.03	-0.09 : 0.03
b_Prevotella_9	0.00	0.00	-0.01 : 0.00
b_Roseburia	-0.05	0.01	-0.07 : -0.02
b_Subdoligranulum	-0.05	0.02	-0.08 : -0.02
b_bmi_tminus	0.83	0.05	0.74 : 0.92
b_s_bw_grams	-0.06	0.04	-0.14 : 0.02
sd_id__Intercept	0.13	0.09	0.01 : 0.32
sigma	0.74	0.03	0.68 : 0.81

Table 11: Random Forest Hyperparameters Per Model

zBMI	Microbiota	Mtry	Sample Fraction
1mo	1m	3	0.8801315
3mo	1m	5	0.7933227
3mo	3m	1	0.7085397
4mo	3m	165	0.3247430
4mo	4m	18	0.8943529
25y	4m	222	0.2006616
6y	6y	12	0.6371634
7y	6y	1	0.2405136
10y	10y	217	0.5188337
125y	10y	169	0.2142654

Table 12: Random Forest Variable Importance for Significant Models. Taxon = Highest identifiable taxon assigned to the amplicon sequence variant ranging from Genus (g) to order (o). SCFA = Whether or not we did identify this taxon as SCFA producer based on our literature review.

Phylum	Taxon	SCFA	Importance	P-value
**Model 1**				
Verrucomicrobia	g___Akkermansia	Yes	0.0219	0.0040
Actinobacteria	g___Corynebacterium_1	No	0.0232	0.0090
Firmicutes	g___Hungatella	No	0.0028	0.0340
Firmicutes	g___Granulicatella	No	0.0129	0.0430
Firmicutes	g___Ruminiclostridium	No	0.0011	0.0519
Firmicutes	g___Lactobacillus	No	0.0134	0.1019
Actinobacteria	g___Actinobaculum	No	0.0006	0.1209
Actinobacteria	g___Senegalimassilia	No	0.0038	0.1239
Firmicutes	g___Gemella	No	0.0059	0.1469
Firmicutes	f___Peptostreptococcaceae	No	0.0009	0.1758
**Model 2**				
Bacteroidetes	f___Bacteroidales_S24-7_group	No	0.0004	0.0190
Actinobacteria	g___Olsenella	No	0.0015	0.0220
Bacteroidetes	g___Bacteroides	Yes	0.0127	0.0290
Bacteroidetes	g___Prevotella_9	Yes	0.0049	0.0350
Firmicutes	g___Peptococcus	No	0.0002	0.0370
Firmicutes	g___Roseburia	Yes	0.0124	0.0410
Firmicutes	g___Dialister	Yes	0.0054	0.0709
Actinobacteria	g___Gordonibacter	No	0.0010	0.0779
Firmicutes	g___[Eubacterium]_eligens_group	No	0.0047	0.0839
Bacteroidetes	f___Prevotellaceae	No	0.0001	0.0839
**Model 3**				
Firmicutes	g___Ruminococcus_2	No	0.0194	0.0430
Bacteroidetes	g___Parabacteroides	No	0.0147	0.0440
Bacteroidetes	g___Prevotella_9	Yes	0.0054	0.0539
Firmicutes	g___Lactobacillus	No	0.0034	0.0549
Firmicutes	g___Roseburia	Yes	0.0161	0.0559
Tenericutes	o___Mollicutes_RF9	No	0.0003	0.0619
Firmicutes	g___Coprococcus_3	Yes	0.0150	0.0649
Bacteroidetes	g___uncultured_bacterium	No	0.0001	0.0649
Firmicutes	g___[Eubacterium]_xylanophilum_group	No	0.0046	0.0809
Bacteroidetes	g___Prevotella_7	Yes	0.0008	0.0849

Table 13: Comparison of accuracy (mean squared error) between 3 machine learning algorithms based on 10x4 fold crossvalidation using all genus relative abundances to predict crossectional zBMI or future zBMI.

Time Microbiota	Time zBMI	Random Forest	Elastic Net	Lasso
1m	1m	0.843	0.833	0.839
1m	3m	0.927	0.924	0.925
3m	3m	0.943	0.94	0.935
3m	4m	0.863	0.856	0.857
4m	4m	0.88	0.918	0.906
4m	2y	0.992	0.944	0.943
6y	6y	0.79	0.777	0.783
6y	7y	1.152	1.12	1.12
10y	10y	0.769	0.81	0.783
10y	12y	1.107	1.211	1.187
**Average**		**0.9266**	**0.9333**	**0.9278**

Table 14: Standardized beta coefficients of the LASSO models for time points where the Random Forest Models were significant. Taxon = Highest identifiable taxon assigned to the amplicon sequence variant ranging from Genus (g) to order (o). SCFA = Whether or not we did identify this taxon as SCFA producer based on our literature review.

Phylum	Taxon	SCFA	Beta
**Model 1**			
Verrucomicrobia	g___Akkermansia	Yes	0.109
Actinobacteria	g___Corynebacterium_1	No	0.070
Firmicutes	g___Subdoligranulum	Yes	0.000
Actinobacteria	g___Bifidobacterium	Yes	0.000
Bacteroidetes	g___Bacteroides	Yes	0.000
Lentisphaerae	f___vadinBE97	No	0.000
Tenericutes	o___NB1-n	No	0.000
Bacteroidetes	f___Porphyromonadaceae	No	0.000
Bacteroidetes	f___Bacteroidales_S24-7_group	No	0.000
Verrucomicrobia	o___Opitutae_vadinHA64	No	0.000
**Model 2**			
Firmicutes	g___Coprococcus_3	Yes	0.099
Bacteroidetes	g___Prevotella_9	Yes	0.051
Firmicutes	g___Family_XIII_UCG-001	No	-0.038
Tenericutes	o___Mollicutes_RF9	No	-0.031
Actinobacteria	g___Senegalimassilia	No	0.031
Firmicutes	g___Dialister	Yes	0.020
Actinobacteria	g___Olsenella	No	0.010
Firmicutes	g___Subdoligranulum	Yes	0.000
Actinobacteria	g___Bifidobacterium	Yes	0.000
Bacteroidetes	g___Bacteroides	Yes	0.000
**Model 3**			
Firmicutes	g___Coprococcus_3	Yes	0.106
Firmicutes	g___Ruminococcus_2	No	-0.088
Firmicutes	g___Dialister	Yes	0.060
Tenericutes	o___Mollicutes_RF9	No	-0.041
Firmicutes	g___Christensenellaceae_R-7_group	No	-0.030
Actinobacteria	g___Senegalimassilia	No	0.023
Bacteroidetes	g___Prevotella_7	Yes	0.013
Bacteroidetes	o___Bacteroidales	No	0.010
Bacteroidetes	g___Alistipes	Yes	-0.006
Firmicutes	g___Subdoligranulum	Yes	0.000
